# Akt Isoforms: A Family Affair in Breast Cancer

**DOI:** 10.3390/cancers13143445

**Published:** 2021-07-09

**Authors:** Alakananda Basu, Christoffer Briggs Lambring

**Affiliations:** Department of Microbiology, Immunology and Genetics, University of North Texas Health Science Center, Fort Worth, TX 76107, USA; ChristofferLambring@my.unthsc.edu

**Keywords:** Akt isoforms, regulation, breast cancer initiation and progression, cell proliferation, autophagy, senescence, metabolism, tumorigenesis, metastasis, AGC kinase

## Abstract

**Simple Summary:**

Breast cancer is the second leading cause of cancer-related death in women in the United States. The Akt signaling pathway is deregulated in approximately 70% of patients with breast cancer. While targeting Akt is an effective therapeutic strategy for the treatment of breast cancer, there are several members in the Akt family that play distinct roles in breast cancer. However, the function of Akt isoforms depends on many factors. This review analyzes current progress on the isoform-specific functions of Akt isoforms in breast cancer.

**Abstract:**

Akt, also known as protein kinase B (PKB), belongs to the AGC family of protein kinases. It acts downstream of the phosphatidylinositol 3-kinase (PI3K) and regulates diverse cellular processes, including cell proliferation, cell survival, metabolism, tumor growth and metastasis. The PI3K/Akt signaling pathway is frequently deregulated in breast cancer and plays an important role in the development and progression of breast cancer. There are three closely related members in the Akt family, namely Akt1(PKBα), Akt2(PKBβ) and Akt3(PKBγ). Although Akt isoforms share similar structures, they exhibit redundant, distinct as well as opposite functions. While the Akt signaling pathway is an important target for cancer therapy, an understanding of the isoform-specific function of Akt is critical to effectively target this pathway. However, our perception regarding how Akt isoforms contribute to the genesis and progression of breast cancer changes as we gain new knowledge. The purpose of this review article is to analyze current literatures on distinct functions of Akt isoforms in breast cancer.

## 1. Introduction

Breast cancer is the second leading cause of cancer-related death, affecting 1 in 8 women in the United States. It is also a common cancer worldwide. It is anticipated that approximately 281,550 cases will be diagnosed, and 43,600 women are expected to die from breast cancer in 2021 (breastcancer.org, 1 May 2021). Based on PAM50 gene expression profiling, breast cancer is categorized in four different subtypes: luminal A, luminal B, HER2-enriched and basal-like [1]. The status of estrogen receptor (ER), progesterone receptor (PR) and epidermal growth factor receptor 2 (HER2) detected by immunohistochemistry is used in the clinic to decide treatment strategy for breast cancer [2]. While luminal A breast cancers are ER-positive, PR-positive and HER2-negative, luminal B breast cancers are ER-positive, PR-negative and HER2-positive. HER2-enriched breast cancers are negative for ER and PR but overexpress HER2 whereas basal-like (BL) breast cancers do not express ER, PR or HER2 and are often used synonymously with triple-negative breast cancer (TNBC).

The phosphatidylinositol 3-kinase (PI3K)/Akt signaling pathway is frequently deregulated in breast cancer [3,4]. PI3K contains a p85 regulatory subunit and a p110 catalytic subunit. In response to growth factors, the lipid kinase activity of class I PI3K becomes activated and generates the second messenger PIP3 from PIP2, resulting in the activation of Akt [5]. The lipid phosphatase activity of the tumor suppressor PTEN, which dephosphorylates PIP3, causes inactivation of PI3K, thereby inhibiting Akt [6]. Although mutations in Akt are rare, activating mutations in the catalytic subunit of p110 PI3K (PIK3CA) have been found in breast cancer [7]. In addition, amplification of HER2 as well as mutations in PTEN could also result in the activation of PI3K/Akt signaling. Mutations in PIK3CA are most common in luminal type of breast cancer [8,9], but have also been found in TNBC [3,10]. HER2 is often amplified in HER2-enriched breast cancer whereas decreased PTEN and increased EGFR expression is associated with TNBC [9]. Reduction in PTEN levels were also associated with lymph node metastases and poor prognosis of breast cancer [11].

Akt belongs to the AGC group of serine/threonine protein kinases and is also known as protein kinase B due to its similarities with protein kinase A and protein kinase C [12,13,14]. It regulates many cellular processes including cell proliferation, cell survival, metabolism and metastasis [15,16]. It is a major mediator of the oncogenic signaling acting downstream of PI3K. There are three members in the Akt family, namely, Akt1(PKBα), Akt2(PKBβ) and Akt3(PKBγ) [17]. Although the overall structure of Akt isoforms is similar, they exhibit redundant and even opposite functions depending on the cellular context. In this review article, we have discussed recent advancements on the understanding of distinct functions of Akt isoforms in breast cancer.

## 2. Structural Heterogeneity and Regulation of Akt Isoforms

Three members of the Akt family Akt1, Akt2 and Akt3 are encoded by distinct genes located in chromosomes 14q32 [18], 19q13 [19] and 1q44 [20], respectively. All three Akt isoforms contain an N-terminal pleckstrin homology (PH) domain, a central kinase domain and a C-terminal regulatory domain that contains a hydrophobic motif (HM) site. While the linker region that tethers the PH to the catalytic domain is divergent, there is considerable sequence homology among these domains [21,22]. 

**Figure 1:**
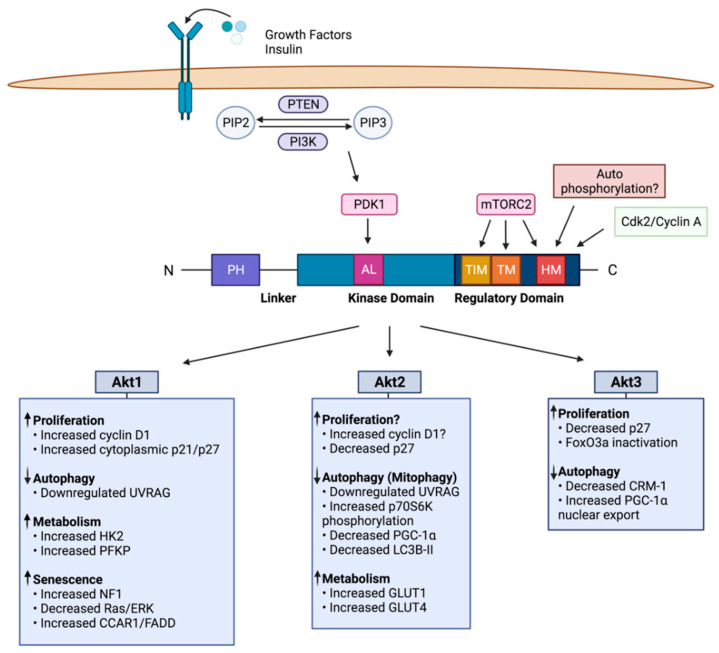
Regulation of cellular processes by Akt isoforms. Phosphorylation of Akt isoforms by upstream kinases triggers distinct cellular responses as indicated by upward and downward arrows. AL—activation loop, TIM—TOR interacting motif, TM—turn motif, HM—hydrophobic motif, PH—pleckstrin homology.

Activity of Akt is regulated by conformational changes and phosphorylation. Akt isoforms can be phosphorylated at approximately 20 different sites and differential phosphorylation of Akt isoforms may be responsible for distinct substrate specificities and their non-redundant functions [23]. Akt is phosphorylated at the conserved activation loop (A-loop or AL), turn motif (TM) and hydrophobic motif (HM) sites. In the absence of a stimulus, the interaction of the PH domain with the kinase domain maintains Akt in an inactive compact structure in the cytosol [24]. When PI3K becomes activated, it phosphorylates phosphoinositides to generate the second messenger phosphatidylinositol (3,4,5)-trisphosphate (PIP3) from phosphatidylinositol (4,5)-bisphosphate (PIP2) (Figure 1). According to the canonical model, PIP3 binds to the PH domain of Akt inducing its membrane recruitment and conformational change which relieves its negative regulation. Phosphoinositide-dependent kinase 1 (PDK1) also contains a PH domain. Thus, the generation of PIP3 facilitates recruitment of PDK1 to the membrane where it phosphorylates Akt at the activation loop (T308, T309, T305 in Akt1, Akt2, and Akt3, respectively) [25]. Mechanistic target of rapamycin complex 2 (mTORC2) phosphorylates Akt at the C-terminal HM site (S473, S474, S472 in Akt1, Akt2, and Akt3, respectively) resulting in full activation of Akt [17,26]. It is believed that in response to insulin or growth factors, mTORC2 phosphorylates Akt at the HM site whereas during DNA damage or stress, DNA-PK is primarily responsible for HM phosphorylation [27]. Several other kinases including mitogen-activated protein kinase (MAPK) [28], integrin-linked kinase (ILK) [29], protein kinase C (PKC)-α [30], PKCβII [31], ataxia telangiectasia mutated (ATM) [32], IκB kinase-κ (IKKε)/TANK-binding kinase 1 (TBK1) [33] IκB kinase-α (IKKα) [34] and cyclin D1 [35] have been implicated in phosphorylating Akt at the HM site. It was suggested that HM site phosphorylation is regulated by autophosphorylation and is independent of mTORC2 but depends on the removal of the PH domain from the kinase domain and phosphorylation at the A-loop by PDK1 [36]. Once activated, Akt translocates to different compartments to transduce signals [37]. 

Akt is phosphorylated at the turn motif (TM) independent of growth factor stimulation of PI3K activity. The TM site is phosphorylated co-translationally by mTORC2 (T450, T451 and T447 in Akt1, Akt2, and Akt3, respectively) and is required for Akt folding, maturation, and stability but does not directly regulate Akt activity [38]. Although mTORC2 mediates phosphorylation of both HM and TM sites of Akt, phosphorylation at these sites have differential effects on Akt activity. HM phosphorylation enhances phosphorylation at the A-loop and increases Akt activity whereas TM phosphorylation induces dephosphorylation of A-loop phosphorylation, thus decreasing Akt activity [39]. A recent study identified additional mTORC2-regulated phosphorylation sites termed TOR interacting motif (TIM) at the C-terminal tail [40]. These sites are conserved among AGC kinases (Akt1-T443, Akt-T444, Akt3-T440) and regulate A-loop phosphorylation by PDK1. Although there are controversies whether mTORC2 directly phosphorylates HM site or not, it can regulate Akt activity by phosphorylating TIM [40].

The C-terminal regulatory tail contains additional phosphorylation sites S477 and S479 (Akt1) which can be phosphorylated in a manner dependent on cell cycle progression [41]. Phosphorylation of these sites by cyclin-dependent kinase 2 (Cdk2/cyclinA) results in Akt activation [41]. S477/S479 sites were also phosphorylated by mTORC2 or DNA-PK in response to insulin or DNA damage, respectively. A correlation between S477/S479 phosphorylation and cyclin A2 expression was found in breast cancer patient samples [41]. Cdk2/cyclin A also phosphorylated Akt2 at S478 and synergized with S474 phosphorylation at the HM site to allosterically activate Akt2 [41]. Recently, it was reported that amino acids 44-46 (DVD) at the PH domain affect C-terminal phosphorylation [42]. Triple mutation of DVD (Asp Val Asp) to GPG (Gly Pro Gly) affected phosphorylation of Akt at S473 and T308 but not at S477 and S479. All three Akt isoforms undergo alternate splicing to generate splice variants with altered C-termini that lack the regulatory HM phosphorylation sites and additional C-terminal phosphorylation sites (Ser477 and Tyr479 for Akt1) [43].

Dephosphorylation of Akt results in inactivation of Akt and termination of Akt signaling. Dephosphorylation of PI(3,4,5)P_3_ and PI(3,4)P_2_ to PI(4,5)P2 and PI(3)P by PTEN [44] and INPP4B (inositol polyphosphate 4-phosphatase type II) [45], respectively results in inactivation of PI3K/Akt. Protein phosphatase 2A (PP2A) and the PH domain and leucine-rich repeat protein phosphatases (PHLPP) can directly dephosphorylate Akt. PP2A dephosphorylates the A-loop (T308 for Akt1) site [46] whereas PHLPP dephosphorylates the HM site [47]. There are two isoforms PHLPP1 and PHLPP2 with distinct specificities towards Akt isoforms [48]. While PHLPP2 dephosphorylates Akt1 and Akt3, PHLPP1 dephosphorylates Akt2 and Akt3.

The activity of Akt is also regulated by additional post-translational modifications, such as methylation and ubiquitination [49,50]. TRAF6 (tumor necrosis factor receptor-associated factor 6)-mediated ubiquitination of Akt was required for its membrane localization, phosphorylation and activation [51]. Both Akt1 and Akt2 were shown to interact with TRAF6 ubiquitin ligase. Methylation of Akt1 at K64 by the histone-lysine N-methyltransferase SETDB1 was essential for the recruitment of TRAF6 and Skp2-SCF ubiquitin ligase to Akt to trigger ubiquitination, membrane recruitment and phosphorylation/activation of Akt [49]. AKT E17K mutant exhibited enhanced methylation and ubiquitination [49,50]. Methylation of Akt2 and Akt3 was much less compared to Akt1 [49]. The mitochondrial E3 ubiquitin protein ligase 1 (Mul1 or MULAN) was shown to interact with active phosphorylated Akt1 and Akt2 to trigger their degradation, thus negatively regulating Akt signaling [52].

## 3. Genetic Alterations of Akt Isoforms

No mutations in the catalytic domain of Akt isoforms were detected in breast cancer specimens. A somatic mutation of Glu17 to Lys (E17K) in the PH domain of *Akt1* gene was shown to change conformation causing plasma membrane localization and constitutive activation of Akt1 [53] but it was only weakly constitutively active [54]. The overall prevalence of Akt1 E17K mutation was 6.3% in breast cancers but it varied with tumor grade (11.1% in grade 1 and 1.9% in grade 3). While *Akt1 E17K* may serve as an oncogene in some luminal breast cancers [55], additional mutations may be required to promote tumorigenesis [17]. Recently, an OncoOmics approach that consisted of genomic alterations, signaling pathways, protein-protein interaction network and protein expression in cell lines and patient-derived xenografts was used to determine breast cancer dependency and *Akt1* was shown to be an essential gene in at least three different OncoOmics approaches [56]. *Akt1 E17K* mutation was restricted to hormone receptor-positive luminal breast cancers [57,58,59]. Similar mutation in Akt2 and Akt3 was rare [3,60]. Based on next-generation sequencing analysis, genetic alteration in *Akt1* (E17K and other pathologic mutations) was significantly enriched in metastatic breast cancer compared to primary breast cancer and Akt1, but not Akt2 or Akt3, was identified as an actionable target [61]. 

It has been reported Akt2 was amplified in 3% of breast cancers [62] and Akt3 is frequently amplified in TNBC [63]. Based on TCGA dataset, Akt3 is most amplified followed by Akt2 and amplification of Akt1 was least among Akt isoforms in breast cancer [64]. A recurrent MAGI3-AKT3 fusion that resulted in constitutive activation of Akt was enriched in TNBC [65]. Akt1 copy gain/high mRNA expression was associated with poor prognosis of basal-like 2 (BL2) breast cancer, a subgroup of TNBC [66].

## 4. Function of Akt Isoforms

Akt was originally discovered as an oncogene. While oncogenes contribute to tumorigenesis by increasing cell proliferation, they must overcome several barriers, including apoptosis and senescence and gain the ability to invade and metastasize to acquire a fully malignant phenotype. There have been numerous studies on the anti-apoptotic function of Akt. Most of the earlier studies have been focused on Akt1. Knockout of individual Akt isoform in mice revealed their distinct functions [67]. In the following section, we have discussed how Akt isoforms regulate various cellular processes that contribute to breast cancer pathogenesis (Figure 1 and Table 1).

### 4.1. Cell Proliferation and Survival

Cell cycle progression depends on the activity of cyclin dependent kinases (CDK) which require cyclins for their activation. Induction of cyclin D activates CDK4/6 which phosphorylates and inactivates tumor suppressor protein Rb to allow cell cycle progression [68]. Cyclin-dependent kinase inhibitors (CKI), such as p21 and p27 encoded by *CDKN1A* and *CDKN1B*, respectively, inhibit CDK2-cyclin E and CDK2-cyclin A complexes halting cell cycle progression [69].

Akt1, but not Akt2, was shown to promote breast cancer cell proliferation by upregulating cyclin D1 and S6 (a downstream target of mTORC1) in IBH-6 and T47D breast cancer cells [70]. A recent study showed that there is cross-regulation between Akt1 and cyclin D1 [35]. Extranuclear membrane-associated cyclin D1 was shown to associate with Akt1 and enhanced Akt1 activity both in vitro and in vivo in response to growth factors and increased cell proliferation [35]. Akt1 gene expression signature positively correlated with cyclin D1 gene expression signature in different subtypes of breast cancer with highest significance in luminal A, luminal B and basal type [35].

There are controversies regarding the role of Akt2 on cell proliferation. Santi et al. reported that Akt2, but not Akt1 or Akt3, played a more prominent role in augmenting cell proliferation in triple-negative breast cancer MDA-MB-231 cells [71]. Silencing of Akt2 induced cell cycle arrest at the G0/G1 phase by downregulating cyclin D1. Another study, however, showed that knockdown of Akt1, but not Akt2, decreased cyclin D1 expression and inhibited MDA-MB-231 cell proliferation [72]. Recently, Akt2-specific nanobodies that bind to the hydrophobic motif of Akt2 and reduce phosphorylation of Akt2 at the HM site were developed [73]. These Akt2 nanobodies inhibited cell cycle progression of MDA-MB-231 cells at the G0/G1 phase by decreasing cyclin D1 levels [73].

The mechanisms by which CKIs, such as p21 or p27 regulate cell cycle progression are complex. p21 and p27 inhibit cell cycle progression when localized in the nucleus. Phosphorylation of p21 at Ser145 and p27 at Thr157/Thr198 alters their localization from the nucleus to the cytosol where they cannot inhibit cell cycle progression [74,75,76,77,78]. Heron-Milhavet et al. showed that Akt1 phosphorylates p21 at T145 and induces its cytoplasmic localization in myoblasts whereas Akt2 interacts with unphosphorylated p21 at the nucleus causing an increase in p21 and cell cycle exit [79]. Phosphorylation of p21 by Akt1 prevented interaction of p21 with Akt2 and localized p21 to the cytosol [79]. Phosphorylation of p21 at S146 by Akt increased its stability in MCF-7 cells and promoted cell survival [78]. Although p21 is believed to inhibit cell proliferation, it could also enhance cell cycle progression at the G1/S phase by promoting the assembly and activation of cyclin D-CDK4/6 [78]. S146-p21 did not interact with or inhibit cyclin E-CDK2, but increased cyclin D1 level [78]. Akt1-mediated phosphorylation, cytoplasmic translocation, and stabilization of Skp2 caused degradation of p27 [80] and accumulation of cytoplasmic T157-p27 correlated with Akt activity in primary breast cancer tissues [75,81]. Thus, Akt1 can increase cell proliferation not only by altering the levels of p21 and p27, but also by inducing their nucleo-cytoplasmic shuttling.

**Table 1:** Function of Akt isoforms.

Akt Isoform	Cell line/Model System	Expression	Phenotype/Function	Potential Mechanisms	Ref
Akt2	MDA-MB-231	Akt2 siRNA	↓ Cell proliferation, ↑ Mitophagy	↓ Cyclin D1, ↑ p27, ↓ mTORC1, ↑ PGC-1⍺	[71]
Akt1	MDA-MB-231	Akt1 siRNA	↓ Cell proliferation, ↑ Apoptosis	↓ Cyclin D1	[72]
Akt2	MDA-MB-231	Akt2 nanobodies	↓ Cell proliferation, ↑ Autophagy	↓ Cyclin D1, ↑ LC3B-II	[73]
Akt1	SKBR3	Akt1 siRNA	↓ Cell proliferation	↑ p27	[80]
Akt1	MDA-MB-231 cell line & xenograft	Akt1 siRNA	↓ Cell proliferation & tumor growth		[82]
Akt2		Akt2 siRNA	No effect on cell proliferation & tumor growth		
Akt3		Akt3 siRNA	↓ Cell proliferation & tumor growth		
Akt3	Mouse mammary tumor C4 cells	Akt3 shRNA	↓ Cell proliferation, ↑ Tamoxifen sensitivity	↓ pErbB2/pErbB3, ↓ Foxo3a, ↑ ERα	[83]
Akt3	MDA-MB-468 & MCF10DCIS xenografts	Akt3 shRNA	↓ TNBC growth in 3D culture & xenografts	↑ p27	[63]
Akt3	3475 (metastatic MDA-MB-231 cell line)	Overexpression of Akt3/-S472	↓ Tumor growth & metastasis, ↑ Apoptosis	↓ ERK, ↑ Bim, ↑ Bax activation	[84]
Akt1	MDA-MB-231	CA-Akt1 (Myr-Akt1)	↓ Autophagy	↓ UVRAG	[85]
Akt2		CA-Akt2 (Myr-Akt2)	↓ Autophagy	↓ UVRAG	
Akt2	* MDA-MB-435	WT Akt2 cDNA	↑ Invasion & metastasis	↑ β1-integrin	[86]
Akt1	Transgenic mice with activated ErbB-2	Activated Akt1 (Akt1-DD)	↑ Cell proliferation, ↑ Tumorigenesis, ↓ Metastasis	↑ Cyclin D1, ↑ Rb phosphorylation	[87]
Akt1	Transgenic mice expressing	Activated Akt1 (Akt1-DD)	↑ Tumorigenesis, ↓ Metastasis	↑ Nuclear ER⍺	[88]
Akt2	ErbB2 or PyVmT	Activated Akt2 (Akt2-DD)	↑ Invasion & metastasis		
Akt1	IGF-IR-overexpressing MCF-10A cell	Akt1 siRNA	↓ Cell proliferation, ↑ EMT, ↑ Migration	↑ ERK activity	[89]
Akt2	monolayer and 3D culture	Akt2 siRNA	↓ Cell proliferation, ↓ EMT, ↓ Migration		
Akt1	MDA-MB-231	CA-Akt1 (Myr-Akt1)	↓ Migration	↑ Palladin phosphorylation (S507)	[90]
Akt1	MDA-MB-231, * MDA-MB-435, SUM-159-PT	CA-Akt1 (Myr-Akt1)	↓ Migration and invasion	↑ HDM2 phosphorylation, ↓ NFAT1	[91]
Akt1	T4-2 cells and mouse xenografts	CA-Akt1	↑ Tumor growth, ↓ Cell migration & invasion	↑ T1462 TSC2, ↓ TSC2, ↓ Rho-GTPase	[92]
Akt2	MCF-7 and * MDA-MB-435 cells	Akt2 siRNA	↓ Migration & invasion	Transactivation of Akt2 by Twist	[93]
Akt1	IBH-6, T47D	shAkt1	↓ Tumor growth, ↑ Migration & invasion	↓ Cyclin D1, ↑ β1-integrin & FAK	[70]
Akt2		shAkt2	↓ Migration, invasion & lung metastases	↓ F-Actin & vimentin	
Akt1	MCF-10A, MCF-7 and BT474 cells	Akt1 siRNA	↑ EMT	↓ miR-200, ↑ Zeb1, ↓ E-cadherin	[94]
	MMTV-cErbB2 mice	Akt1 knockout	↑ Migration & invasion		
Akt1	MCF-7, BT-474, MDA-MB-231, SKBR3	Akt1 siRNA	↑ EMT and invasion	↑ EGFR/ERK activity, >↑ Nuclear β-catenin	[95]
Akt2	MDA-MB-231, MDA-MB-468, MCF-7,	Akt2 siRNA	↓ Non-CSC reversion, ↓ CSC survival	↓ Twist, ↓ mTOR	[96]
	SKBR-3 cells & MDA-MB-231 xenografts		↓ EMT and invasion		
Akt1	MCF-10A, p53-null background, MCF-7	Akt1 E17K	↑ Cell growth, ↓ Migration & invasion	↓ Zeb1, ↑ E-cadherin	[97]
Akt2		Myr-Akt2		↑ β-catenin transcription, ↓ E-cadherin	
Akt3	MDA-MB-231 BO cells & xenografts	Akt3 siRNA	↑ Migration, invasion and bone metastasis	↑ HER2 and DDR kinase, ↓ CTGF	[98]
Akt1	Transgenic MMTV-ErbB2 mice	*Akt1* knockout	↓ Cell proliferation, ↓ Tumor growth	↓ p21, p27 & cyclin D1	[99]
	Monolayer & 3D culture of MEC		↓ Cell migration and Lung metastases	↓TSC2 phosphorylation (S939), ↓CXCL-16	
Akt1	MMTV-ErbB2 & MMTV-PyMT mice	*Akt1* knockout	↓Tumorigenesis, ↑ Invasion, ↓Lung metastases	↓Cyclin D1	[100]
Akt2		*Akt2* knockout	↑ Tumorigenesis, ↓metastases	↑ Cyclin D1	
Akt3		*Akt3* knockout	No effect on tumorigenesis or lung metastases		
Akt1	Immortalized MEC	Akt1 and vAkt1	↑ Invasion	↓ MMP2 degradation	[101]
Akt1	IBC cells-SUM149	Akt1 siRNA	↓ IBC cell invasion, no effect in nIBC cells	↓ RhoC GTPase activity	[102]
Akt2	nIBC cells-MDA-MB-231 & * MDA-MB-435	Akt2 siRNA	↓ nIBC cell invasion, no effect in IBC cells		
		Akt3 siRNA	↓ Survival of IBC but not nIBC cells		
Akt3			No effect on invasion in IBC & nIBC cells		
Akt1	HER2-enriched MMTV-NIC mouse model	Cell-autonomous Akt1 deletion	↓ Tumor development, No effect on lung metastasis		[103]
Akt2		Cell-autonomous Akt2 deletion	↓ Tumor development completely		
Akt1	HER2-enriched MMTV-ErbB2 mouse model	Systemic Akt1 deletion	↓ Tumor growth, ↓ metastasis completely		
Akt2		Systemic Akt2 deletion	↑ Tumor growth, ↑ metastasis markedly	↑ Insulin, ↑ ErbB2, ↑ Akt1 activity	
Akt1	Luminal B MMTV-PyMT mouse model	Cell-autonomous Akt1 deletion	No effect on metastasis		
Akt2		Cell-autonomous Akt2 deletion	No effect on metastasis		
Akt1	Luminal B MMTV-PyMT mouse model	Systemic Akt1 deletion	Improved tumor-free survival, ↓ lung metastasis	↓ Survival & mobilization of neutrophil	
Akt2		Systemic Akt2 deletion	No effect on survival or lung metastasis	↑ Akt1 activity	

* Please note that although MDA-MB-435 cells were originally isolated as breast cancer cells, based on cell authentication they are now characterized as melanoma cells.

Akt2 on the other hand was shown to interact with Prohibitin 2/Repressor of Estrogctivator (PHB2/REA) which has been implicated in transcriptional repression of myogenesis in estrogen-dependent cancers like breast cancer [104,105]. Using N-terminal truncation, Heron-Milhavet et al. showed that Akt2 directly binds p21 on a 27 amino acid sequence at 410-437 in the C-terminal domain of Akt2 leading to cell cycle arrest and myogenic differentiation [106]. Santi et al., however, reported that Akt2 increased cell proliferation in MDA-MB-231 cells by reducing p27 levels [71]. Akt2 had no effect on T157/T198 phosphorylation which has been associated with cytoplasmic localization, but Akt2 knockdown enhanced phosphorylation of p27 at Thr187 due to a decrease in Skp2 which targets T187-p27 for degradation via the ubiquitin proteasome-mediated pathway [71]. In addition, miRNA-615 which directly targets Akt2, inhibited MDA-MB-231 cell proliferation by upregulating p21 and p27, implicating a role for Akt2 in cell proliferation [107]. In contrast, Toulany and colleagues showed that knockdown of Akt1 and to some extent Akt3, but not Akt2, inhibited cell proliferation and tumor growth in K-Ras-mutated MDA-MB-231 cells [82]. The reason for the contradictory results from different laboratories with the same cell line is not clear except Santi et al. [71] and Bai et al. [107] depleted Akt2 by siRNA or miRNA and monitored overall cell growth using MTT assay whereas Toulany et al. selected shRNA-transfected MDA-MB-231 cells and assessed clonogenic activity in vitro and tumor growth in vivo [82]. 

It has been reported that Akt3 can contribute to the proliferation of ErbB2 (HER2)-positive breast cancers which express low levels of ERα and contribute to endocrine resistance [83]. Knockdown of Akt1 and Akt3 in ErbB2-positive Erα-negative mouse mammary tumor C4 cells caused a substantial decrease in cell proliferation whereas Akt2 knockdown had only a modest effect [83]. Knockdown of Akt3 induced ERα via FoxO3a and restored sensitivity to tamoxifen. However, the most significant effects of Akt3 in terms of growth and proliferation of breast cancer have been shown in TNBC. Search for potential targets of TNBC using short hairpin RNA screen of protein kinases identified Akt3 as a regulator of TNBC cell growth [63]. Silencing of Akt3 with shRNA inhibited three-dimensional (3D) spheroid growth and TNBC xenografts in nude mice via upregulation of p27 [63]. Akt3 is also a target of miRNA-433 which inhibits cell proliferation and cell survival of TNBC cells by downregulating Akt3 [108]. Analysis of clinical samples of breast cancer tissues revealed an inverse relationship between miRNA-433 and Akt3 mRNA, the latter being much higher in breast cancer patient samples compared to normal breast tissues [108]. A splice variant of Akt3 that lacks Ser472 phosphorylation site induced apoptosis and suppressed TNBC 3475 cell (a metastatic subline of MDA-MB-231 cells) growth in vivo by upregulating pro-apoptotic Bcl-2 family member BIM but had no effect on the proliferation of these cells [84].

### 4.2. Autophagy

Autophagy is a process of self-cannibalism that allows cells to survive under nutrient-deprived or stressful conditions [109]. Autophagy also plays an important role in breast cancer development and progression [110]. Although autophagy is considered a survival mechanism [111] it can also cause cell death and tumor suppression [112,113]. mTORC1, which functions downstream of Akt, is considered the master regulator of autophagy [109]. There are different types of autophagy depending on the organelles that are affected. For example, bulk degradation of cellular organelles is known as macroautophagy and often used synonymously with autophagy whereas selective degradation of specific organelles, such as mitochondria is known as mitophagy.

Santi et al. reported that knockdown of Akt2 in MDA-MB-231 cells attenuated phosphorylation of p70 S6 kinase (p70S6K) at Thr389 site, indicating inhibition of mTORC1 activity [71]. Akt2 was shown to physically associate with mitochondria and prolonged knockdown of Akt2, but not Akt1 or Akt3, induced mitochondrial biogenesis by upregulating peroxisome proliferator-activated receptor coactivator-1α (PGC-1α) and ultimately led to cell death by mitophagy. Thus, Akt2 promoted survival of MDA-MB-231 cells by protecting against mitophagy. However, knockout of individual Akt isoform in mice revealed that Akt3 but not Akt1 promotes mitochondrial biogenesis. Ablation of Akt3 induced autophagy by stabilizing nuclear export protein CRM-1 which exports PGC-1α from the nucleus to the cytoplasm causing a decrease in PGC-1α-dependent gene expression and mitochondrial content [114]. Yang et al. on the other hand showed that both Akt1 and Akt2 inhibited autophagy in MDA-MB-231 cells by downregulating ultraviolet irradiation resistance-associated gene (UVRAG) which is involved in the autophagosome formation and maturation during autophagy (macroautophagy) and sensitized cells to UV irradiation by inhibiting cell proliferation [85]. The effect of Akt1 on UVRAG was independent of Akt1 and mTORC1 activity. Interestingly, while overexpression of Akt1 protected against apoptosis induced by UV irradiation, it decreased cell proliferation by inhibiting autophagy and overcame the anti-apoptotic effect of Akt1. Akt2 also attenuated autophagy by inhibiting the function of the transcription factor EB (TFEB) which regulates the expression of several autophagy-related genes, including LC3B [73]. Phosphorylation of TFEB by mTORC1 causes its cytoplasmic localization and inhibition of its transcriptional activity [109]. The Akt2-specific nanobody Nb8 which was shown to inhibit Akt2 activity and cell cycle progression in MDA-MB-231 cells caused a decrease in TFEB phosphorylation and upregulation of LC3B-II, suggesting Akt2 indirectly regulates TFEB via mTORC1 [73]. Akt3-derived circRNAs hsa_circ_0000199, which has been associated with the clinical pathology of TNBC, was shown to mediate its function by downregulating miR-206 and miR-613 [115]. Silencing of hsa_circ_0000199 decreased cell proliferation, migration and invasion and increased autophagy in TNBC cells and these effects were reversed by inhibitors of miR-206 and miR-613 [115].

Several studies performed in other cell lines suggested additional involvement of Akt isoforms in autophagy. Both Akt1 and Akt2, but not Akt3, were shown to interact with the lysosomal protein pleckstrin homology domain-containing family F member 2 (Phafin2) and knockdown of these isoforms inhibited induction of autophagy. However, only re-introduction of Akt2 but not Akt1 restored autophagy. Interaction of Akt2 with Phafin2 following initiation of autophagy caused accumulation of the complex in the lysosomes where the interaction of Phafin2 with PtdIns(3)P facilitated autophagy induction [116]. The same group showed that vaccinia-related kinase (VRK)-2, a member of the VRK family of serine/threonine protein kinases, also interacts with Akt1 and Akt2 but not Akt3 and accumulates phosphorylated Akt in the lysosomes [117]. Increase in lysosomal Akt activity was important for lysosomal acidification, activation of lysosomal hydrolases and completion of the autophagy process. The orphan nuclear receptor TR3 or Nur77 was shown to induce autophagy via the mitochondrial signaling pathway [118]. Phosphorylation of TR3 by Akt2 retained it in the nucleus and inhibited autophagic cell death in response to 1-(3,4,5-trihydroxyphenyl)nonan-1-one (THPN) in gastric cancer SGC7901 and cervical cancer Hela cells [119]. Interestingly, while phosphorylation of TR3 at Ser533 inhibited autophagy, phosphorylation of TR3 at Ser351 by Akt1 inhibited apoptosis [119]. Recently, it has been shown that Akt1 and Akt3, but not Akt2, is required for the increase in lysosomal vacuolar H^1^-ATPase (V-ATPase) activity in response to amino acid starvation [120]. 

### 4.3. Cellular Senescence

Senescence is defined as prolonged cell cycle arrest and the proteins that inhibit cell cycle progression also play important roles in regulating senescence [121,122]. Since phosphorylation of the tumor suppressor protein Rb by CDK4/6 is required for cell cycle progression, an increase in CDK4/6 inhibitor p16/INK4 which inhibits Rb phosphorylation can induce senescence by causing cell cycle arrest [123]. On the other hand, the CDK2 inhibitor p21, a transcriptional target of the tumor suppressor protein p53 which halts cell cycle in response to DNA damage or cellular stress, can trigger senescence [124]. While replicative senescence is a normal process of aging, premature senescence could be induced by hyperactivation of oncogenes or cellular stress to suppress tumorigenesis.

The oncogenic function of Akt could be counteracted by the induction of cellular senescence. Using Akt1/Akt2 double knockout (DKO) mouse embryo fibroblasts it was shown that ablation of Akt1/Akt2 could inhibit replicative senescence as well as premature senescence induced by oxidative stress and Ras oncogene [125]. Activation of Akt induced premature senescence by inhibiting its downstream target FoxO3a transcription factor which scavenges ROS via induction of Sesn3. Chemotherapeutic agents are known to induce premature senescence. Constitutive activation of Akt1 inhibited doxorubicin-induced senescence but appears to augment tamoxifen-induced senescence in breast cancer MCF-7 cells [126]. All three constitutively-active (CA) Akt isoforms were shown to induce senescence in telomerase reverse transcriptase (TERT)-immortalized BJ human fibroblasts (BJ-T) and Akt1 had the most pronounced effect on the induction of senescence [127]. Activated Akt induced senescence by enhancing mTORC1-mediated translation of p53 and increase in its target p21 [127]. Transcriptome and metabolic profiling was utilized to identify the Akt-induced senescence network and the clinical relevance was determined by analyzing TCGA data of fourteen different cancer types, including breast adenocarcinoma [128]. The tumor suppressor protein neurofibromin 1 (NF1) was shown to maintain Akt-induced senescence via suppression of Ras/ERK signaling [128]. In addition, cycle and apoptosis regulator 1 (CCAR1) which is downregulated in breast cancers and FADD which exerts pro-apoptotic activity in breast cancer were shown to mediate Akt-induced senescence [128]. Overexpression of Akt1 in MDA-MB-231 cells was shown to counteract the effects of the chemokine receptor CXCR2 on breast cancer cell growth, metastasis and chemoresistance by inducing senescence [129].

### 4.4. Metabolism

Altered metabolism is one of the hallmarks of cancer. The most well-known example of metabolic reprogramming in terms of cancer, the Warburg effect, results in increased glucose uptake and elevated lactate production. However, there are other aspects of metabolic regulation that are abnormal in cancer cells. Key enzymes such as succinate dehydrogenase and pyruvate kinase are associated with multiple steps in the tumorigenic process [130]. *p53* and *Myc*, which are among the most commonly mutated genes in a variety of cancers, are master regulators of metabolism and through gain of function mutations contribute to cancer progression and metabolic reprogramming [131]. 

Akt isoforms have been studied for their ability to affect metabolic regulation in cancer cells. Oncogenic Akt activation plays a major role in meeting the metabolic needs of the tumorigenic process. At a broad level, Akt plays a role in metabolic function in breast cancer through the phosphorylation of glycogen synthase kinase 3 (GSK3) at Ser9 [131]. This phosphorylation inhibits GSK3β resulting in a decrease of its kinase activity on its multiple downstream targets like β-catenin. Upon GSK3β inhibition by Akt, normal effector function of β-catenin is restored, and β-catenin can undergo normal nuclear translocation. In breast cancer, overexpression of β-catenin has been shown to affect lipid metabolism via differential protein expression. Using MCF-7 cells with an endogenous knockdown of β-catenin Vergara et al. showed alterations in metabolic processes including the tricarboxylic acid cycle (TCA) and lipid metabolism. After β-catenin knockdown a decrease in expression of acetyl-CoA carboxylase, ATP-citrate lyase, and monoacyl glycerol lipase were all seen, suggesting a significant role in β-catenin function in breast cancer cells [132]. 

Altered glucose metabolism is a common hallmark of cancer and it has been shown that constitutively active Akt can promote aerobic glycolysis as well as increase both glycolytic rate and glucose uptake [133,134]. Immunohistochemical analysis of breast cancer specimens by Schmidt et al. showed a correlation between pAkt and glucose transporter 1 (GLUT1) expression in breast cancer specimens, suggesting a role for Akt in the glycolytic phenotype [135]. Knockdown of GLUT4 was shown to induce metabolic reprogramming and decrease viability of breast cancer MCF-7 and MDA-MB-231 cells, and both GLUT1 and GLUT4 have been suggested as prognostic and therapeutic targets in breast cancers [136,137]. Akt2 specifically, has been implicated in most studies involving glucose transporters. Beg et al. recently provided evidence that linked Akt2 to both GLUT1 and GLUT4 translocation in adipocytes [138]. Using a method developed by Kajno et al. [139], they showed that Akt2 T309 phosphorylation by PDK1 was responsible for GLUT4 translocation, while both T309 and S474 phosphorylation were required for GLUT1 translocation to the plasma membrane of proliferative cells [138]. These actions of Akt2 can be partly responsible for increased glucose uptake and the reprogramming of metabolism seen in breast cancer.

Akt isoforms have also been implicated in the glycolysis pathway through modulation of regulatory proteins. In MCF-7 cells, Akt1 was shown to interact with hexokinase 2 (HK2), which phosphorylates glucose to glucose-6P [140]. This relationship shows Akt1′s involvement in a critical regulatory step that blocks glucose from leaving the cell due to conformational changes from hexokinase phosphorylation resulting in increased glycolysis by breast cancer cells. Phosphofructokinase 1 (PFK1) is another important regulatory enzyme in glycolysis, which catalyzes the ATP-dependent conversion of fructose-6P to fructose-1,6-BP. The platelet form of PFK1 (PFKP) is overexpressed in breast cancer cells and has been associated with increased glycolytic efficiency [141,142]. Lee et al. showed a relationship between Akt1 and PFKP in breast cancer cells. Knockdown/inhibition of Akt1 resulted in a reduced half-life of PFKP, while Myr-Akt1 resulted in prolonged half-life of PFKP [143]. This suggests that Akt1 plays a pro-tumorigenic role via manipulation of PFK1. 

Another attribute of breast cancer is alterations in lipid synthesis. Akt has been shown to have multiple effects on tumorigenic lipid synthesis. Little is known about oncogenic Akt isoform specificity in breast cancer in terms of lipid metabolism, but at a homeostatic level Akt1/2/3 have different functions in mammary gland lipid biosynthesis. For example, constitutively-active Akt1 in transgenic mouse mammary glands mediates lipid accumulation during pregnancy, but mostly general Akt studies have made the connection between Akt and lipid metabolism in breast cancer [144]. Akt can directly affect de novo lipid synthesis through interaction with ATP citrate lyase (ACLY). Akt phosphorylates ACLY on S454 resulting in its activation leading to increased production of cytosolic acetyl-CoA which is in turn used in multiple metabolic reactions including sterol and fatty acid synthesis [145]. ACLY is overexpressed in breast cancer and its presence has been suggested as a diagnostic marker for recurrence and possible chemotherapy resistance [146]. While not shown directly in breast cancer yet, Wei et al. showed a link between ACLY and the PI3K-Akt pathway. Utilizing knockdowns of various ovarian cancer cells, they showed that a decrease of ACLY resulted in inhibition of PI3K-Akt possibly through inhibition of p-Akt [147]. 

Akt also acts on sterol regulatory element-binding protein (SREBP-1/-2) transcription factors and fatty acid synthase (FASN), which are involved in different aspects of fatty acid and sterol synthesis [148]. SREBP transcription factors act on promoter regions of genes responsible for lipogenesis and NADPH production [15]. Bao et al. showed SREBP-1 upregulation in breast cancer and in vitro knockout of SREBP decreased migration and invasion of breast cancer cell lines [149]. Recently Yi et al. demonstrated that in multiple cancer cell lines, including MDA-MB-231, oncogenic activation of Akt protected against ferroptosis via SREBP lipogenesis [150]. FASN is involved in the synthesis of long chain fatty acids and is upregulated in multiple breast cancers [151,152]. Xu et al. were able to show that knockdown of FASN not only affects the metabolic profile of breast cancer lines, but also led to decreases in migration of SK-Br-3 cells [151]. FASN inhibition in MCF-7 and BT-474 cells leads to downregulation of Akt and thus overall breast cancer survival [153]. Akt isoform specificity has yet to be extensively studied in breast cancer in relation to FASN, although prostate cancer studies have revealed a relationship between Akt3 and FASN involving the two as downstream effectors of peroxisome proliferator-activated receptor gamma (PPARG) activation [154].

NADPH is a key component of homeostatic lipogenesis and its altered expression is evident in multiple cancers. NADPH has two-fold metabolic functions; fatty acid synthesis involving acetyl-CoA and providing reducing components for multiple cellular processes [155]. Akt has been shown to affect NADPH levels through alteration of the pentose phosphate pathway (PPP), the major source of cytosolic NADPH, through an mTORC1-SREBP axis [156]. Akt has also been shown to affect NADPH production by directly phosphorylating NAD kinase (NADK) and in oncogenic PI3K breast cancer cell line T47D, Akt phosphorylation of NADK facilitated anchorage-independent cell growth [157]. Cui et al. showed that NADPH was involved in apoptosis of breast cancer cells. In multiple breast cancer cell lines, FAS inhibition leading to NADPH accumulation was responsible for apoptosis [158]. These findings together show another mechanism by which oncogenic Akt signaling can contribute to the broad metabolic reprogramming responsible for meeting the needs of developing cancers. NADPH replenishment is a critical step in metabolic functions and understanding the link between Akt and its isoforms and their specific effects on this process could lead to novel therapeutic targets in breast cancer therapy.

### 4.5. Tumor Growth, Invasion and Metastasis 

It is well established that PI3K/Akt signaling is frequently deregulated in breast cancer and plays critical roles in tumorigenesis. While Akt contributes to tumor growth by increasing cell proliferation or decreasing cell death, metastatic dissemination of cancer cells from the primary tumor site to a secondary site involves several steps, including epithelial-to-mesenchymal transition (EMT), invasion, intravasation into the blood vessels, anoikis resistance, and extravasation [159]. In this section, we discuss how Akt isoforms regulate tumor growth and various processes leading to metastasis.

Several studies showed that Akt1 and Akt2 have opposite effects on tumor initiation and tumor progression. Arboleda et al. reported that overexpression of active Akt2, but not Akt1 or Akt3, enhanced invasion through collagen IV matrix via upregulation of β1-integrin in several breast cancer cells and increased metastasis of MDA-MB-435 xenografts [86] (Table 1). Using a transgenic mouse model, Muller and co-workers demonstrated that mammary-specific expression of activated Akt1 accelerated ErbB2-induced mammary tumorigenesis by enhancing cyclin D1 and cell proliferation but suppressed lung metastases [87], suggesting Akt1 had opposite effects on tumor growth and metastases. Activated Akt2 in the same model system did not affect tumor development but caused a substantial increase in lung metastases [88], supporting the notion that Akt1 and Akt2 have distinct effects on tumor initiation versus tumor progression. A similar conclusion was reached by several different laboratories, although the mechanism by which Akt1 and Akt2 regulated invasion and metastasis differed. When basal-like mammary epithelial MCF-10A cells were stimulated by overexpressing insulin-like growth factor-I receptor (IGF-IR), Akt1 downregulation enhanced EMT and cell migration via activation of ERK whereas Akt2 downregulation suppressed EMT and inhibited migration in 3D cell culture [89]. Palladin, an actin-associated protein, was identified as a specific substrate for Akt1 and not Akt2 and was shown to inhibit Akt1-mediated cell migration in breast cancer cells [90]. Ectopic expression of constitutively-active Akt1 in TNBC cells inhibited invasion and migration by phosphorylating HDM2 which triggered degradation of the transcription factor NFAT [91]. Introduction of constitutively-active Akt1 in HMT-3522 T4-2 breast cancer cells enhanced cell proliferation and survival and promoted tumor growth but inhibited invasion and motility by inducing phosphorylation (T1462) and degradation of TSC2 which regulates cell adhesion and migration via Rho-GTPase [92]. Twist, a transcription factor for EMT [93], was shown to mediate invasion in MCF-7 and MBA-MB-453 cells by transactivating the Akt2 promoter and there was a correlation between Akt2 and Twist expression in late-stage breast cancers [160]. Knockdown of Akt1 increased cell migration and invasion by upregulating β1-integrin & focal adhesion kinase (FAK) whereas Akt2 knockdown inhibited migration and invasion by decreasing F-actin and vimentin in T47D and IBH-6 cells [70]. Moreover, based on the analysis of invasive breast cancer samples in TCGA dataset, Akt2, but not Akt1, was associated with worse clinical outcome [70].

A balance between Akt1 and Akt2 decided the invasiveness and metastasis of primary and metastatic breast cancers by differential regulation of miR-200 [94]. Ablation of Akt1, but not Akt2 or Akt3, promoted EMT in MCF-10A cells and increased invasiveness in MMTV-cErb-B2 mice via downregulation of miR-200 which increased Zeb1 and decreased E-cadherin expression. In contrast, Akt2 decreased the abundance of miR-200 in the absence of Akt1, suggesting that the status of both Akt1 and Akt2 is important to determine the ultimate outcome on invasion and metastasis [94]. Li et al. reported that Akt1 knockdown promoted EMT and invasion of breast cancer cells by dephosphorylating and inactivating PIKfyve which caused sustained activation of EGFR and ERK signaling resulting in β-catenin nuclear accumulation [95]. The activation of cancer-activated fibroblasts (CAF), which facilitates invasion of epithelial cells, required Akt2 which was activated by Snail and was distributed in polarized cells that were more abundant in the area of invasion in human breast tumor tissues [161]. Cancer stem cells (CSC) can lead to tumor initiation and progression as well as metastasis [162]. Knockdown/inhibition of Akt2 inhibited metastatic potential of CSC and non-CSC by inhibiting the expression of *TWIST* or *mTOR* and high levels of Akt2 could be detected in circulating tumor cells in orthotopic mouse models [96]. Akt1 E17K mutation in p53 null background inhibited cell migration and invasion in MCF-10A cells by decreasing ZEB1 which caused an increase in E-cadherin and reversal of EMT whereas Akt2 had opposite effect on E-cadherin [97]. While most of the studies have examined the involvement of Akt isoforms in lung metastases, Hinz et al. showed that Akt3 but not Akt1 or Akt2 activity is elevated in a subline of MDA-MB-231 cells that metastasize to bone (MDA-MB-231 BO) and knockdown of Akt3 increased migration, invasion, and bone metastasis via activation of HER2 and discoidin domain receptor (DDR) kinases and downregulation of the TGFβ/CTGF (connective tissue growth factor) axis [98].

Many molecules/compounds have been shown to induce migration, invasion and metastasis via the Akt2 signaling pathway. WDR26, a WD40 protein that is overexpressed in highly malignant breast cancers and is associated with poor survival of breast cancer patients, selectively bound to Akt2 and not Akt1 [163]. In ER-positive breast cancers, there was a negative correlation between miR-124 and Akt2 expression and overexpression of miR-124 inhibited E2-induced cell proliferation, migration and invasion by downregulating Akt2 [164]. UCH-L1 (ubiquitin C-terminal hydrolase), a deubiquitinase, was shown to specifically interact with and activate Akt2 in MCF-7 breast cancer cells to promote invasion but did not affect cell proliferation [165]. Metformin inhibited cell migration and invasion of several breast cancer cells and metastasis of MDA-MB-231 xenografts by upregulating miR-200c which negatively regulated Akt2 expression [166]. Dietary fatty acids, such as linoleic acid and oleic acid were also shown to promote migration and invasion in breast cancer cells via Akt2 [167,168]. 

Several molecules also induced invasion and metastasis by inhibiting Akt1. The chemokine receptor CXCR2 promoted breast cancer cell migration, invasion, and metastasis by suppressing Akt1 [129]. The tumor suppresser, 12-*O*-Tetradecanoyl phorbol-13-acetate (TPA)-inducible sequences 21 (TIS21), an ortholog of B-cell translocation gene 2 (BTG2), inhibited TNBC cell growth and invasion via activation of Akt1 and not Akt2 [169]. A recent study showed that Rho GTPase activating protein 29 (ARHGAP29) interacted with Akt1 and knockdown of ARHGAP29 decreased invasion of breast cancer cells [170]. While ARHGAP29 knockdown downregulated Akt1, the ratio of phosphorylated Akt1 to total Akt1 remained unchanged.

In contrast to most of the published reports that suggest an anti-metastatic function of Akt1, several studies support a pro-metastatic function of Akt1. Ablation of Akt1 in mice not only inhibited ErbB2-induced mammary tumorigenesis and delayed tumor growth by inhibiting phosphorylation of TSC2 at Ser-939, but also decreased lung metastases and reduced migration of mammary epithelial cells both in 2D and 3D cell culture via induction and secretion of CXCL16 [99]. Another study reported that knockout of Akt1 in mice inhibited, Akt2 accelerated, and Akt3 had little effect, on the induction of polyoma middle T (PyMT) and ErbB2-driven mammary carcinogenesis [100]. However, while knockout of Akt1 increased invasiveness of ErbB2/Neu-induced tumors, individual knockout of either Akt1 or Akt2 in PyMT mice inhibited metastasis, suggesting a lack of correlation between invasiveness and metastatic potential in this model system [100]. Akt1 induced invasion of immortalized mammary epithelial cells by preventing degradation of matrix metalloprotease (MMP)-2 by the proteasomal pathway [101]. PTEN expression is lost in inflammatory breast cancer (IBC) SUM149 cells [102]. Knockdown of Akt1 inhibited invasion of IBC cells by inhibiting phosphorylation of RhoC GTPase, a substrate for Akt1, but had no effect on non-IBC (nIBC) cells whereas Akt2 knockdown inhibited invasion of nIBC cells but had no effect on the invasiveness of IBC cells [102]. Akt3 knockdown did not affect invasion of either IBC or nIBC cells. On the other hand, Akt1 and Akt2 knockdown had no effect on cell proliferation and apoptosis of IBC cells whereas depletion of Akt3 decreased survival of IBC but not nIBC cells by inducing apoptosis [102]. *Akt1* was identified as one of the driver genes frequently mutated in patients with hormone receptor-positive/HER2-negative metastatic breast cancer [171]. Moreover, analysis of patient samples identified Akt1 as a major contributor of metastatic lymph node involvement which is a risk for breast cancer progression [172]. Akt1 was highly expressed in ER-positive recurrent breast cancers and negatively affected overall survival of breast cancer patients [172]. 

A recent study revealed that cell autonomous versus systemic deletion of Akt1 and Akt2 had a major impact on the function of Akt1 and Akt2 on tumorigenesis and metastasis [103]. Mammary gland-specific cell-autonomous Akt1 deletion inhibited tumor growth but not metastasis. However, systemic deletion of Akt1 but not Akt2 inhibited lung metastasis through impairment of mobilization and survival of tumor-associated neutrophils and neutrophil-specific deletion of Akt1 suppressed metastasis. On the other hand, while systemic or germline Akt2 deletion did not inhibit or enhance tumorigenesis, cell-autonomous Akt2 deletion prevented tumorigenesis by inhibiting ErbB2 expression in the mammary gland. Systemic Akt2 deletion increased circulating insulin levels which caused hyperactivation of Akt1 and possibly Akt3, and maintained ErbB2 expression, thus interfering with the ability of Akt2 deletion to inhibit tumorigenesis at an early stage, but enhanced ErbB2-induced metastasis. 

## 5. Discussion

The importance of the Akt signaling pathway in the development and progression of breast cancer cannot be overstated. It is now well recognized that Akt isoforms play distinct roles in various cellular processes. Most early studies were focused on Akt1 and this isoform was associated with most of the oncogenic functions of Akt. It was then realized that Akt1 and Akt2 have opposite roles in breast cancer initiation and progression. Akt1 was shown to promote tumor initiation by enhancing cell proliferation, cell survival and tumor growth, but it inhibited tumor progression [70,89,90,94,95]. In contrast, Akt2 facilitated tumor progression by increasing cell migration, invasion and metastasis [70,88,89,96,100,160]. Akt2 was also associated with worse clinical outcome and was considered a worthwhile target for breast cancer therapy [70]. Although Akt3 expression was believed to be restricted to neuronal cells, it is now known that it can also contribute to breast cancer, especially in TNBC [63,82,84,98,102]. 

A consensus regarding isoform-specific functions of Akt, however, could not be reached even within a particular subtype of breast cancer (Table 1). While knockdown of Akt1 in breast cancer cell lines caused an increase in cell migration and invasion [70,89,90,94,95], genetic ablation of Akt1 in mouse models of mammary tumor decreased tumor metastasis [99,100]. However, hyperactivation of Akt1 also led to decrease in metastasis both in vitro and in vivo [87,88,90,91,92]. Likewise, most studies are consistent with the involvement of Akt2 in migration, invasion and metastasis but there are controversies regarding its involvement in cell proliferation and tumorigenesis. Akt2 was reported to decrease [100], increase [71,73,89] or have no effect on cell proliferation and tumor growth [82,88]. Similarly, there are contrasting reports whether depletion of Akt3 increases migration, invasion, and bone metastasis [98], decreases cell proliferation and tumor growth [63,82,83], or has no effect on tumorigenesis and lung metastasis [100].

Cellular context plays a major role in deciding the function of Akt isoforms. The environment in live animal is much more complex compared to cultured cells. Ectopic expression of Akt isoforms may have altered localization compared to endogenous Akt isoforms. On the other hand, while germline knockout of Akt isoforms results in complete ablation of Akt isoforms, knockdown of Akt by siRNA may reduce but may not completely eliminate the abundance of Akt isoforms. However, complete ablation of Akt isoforms may lead to compensatory increase in other signaling pathways. Moreover, since Akt isoforms can regulate each other, the status of Akt isoforms in a particular cell type may influence their function. It is, however, hard to reconcile why the function of Akt isoforms vary even with the same cell line. For example, while some studies suggested knockdown of Akt2 decreased proliferation of MDA-MB-231 cells [71,73,107], other studies suggested knockdown of Akt1 and Akt3, but not Akt2, decreased MDA-MB-231 cell growth [63,82]. The threshold of Akt activity such as hyperactivation of Akt by certain stimulus (e.g., oxidative stress) or overexpression of CA-Akt may have different consequences compared to physiological activation of Akt isoforms. For example, while activation of Akt promotes tumorigenesis by increasing cell proliferation and survival (Table 1), hyperactivation of Akt by reactive oxygen species could suppress tumorigenesis by inducing cellular senescence [125].

A recent study revealed a completely different picture of how Akt1 and Akt2 contribute to tumor development and metastasis based on systemic versus autonomous deletion of Akt1 and Akt2 [103]. Systemic Akt1 deletion inhibited lung metastases whereas systemic Akt2 deletion enhanced mammary tumorigenesis and metastasis at least in HER2-enriched and luminal B mouse models of breast cancer [103]. Moreover, Akt1 was recently identified as one of the driver genes more frequently mutated in hormone receptor-positive metastatic breast cancer [171] and contributed to metastatic lymph node involvement [172].

## 6. Conclusions

In summary, our notion about which Akt isoform to target for cancer therapy is changing. Most studies are consistent with the roles for Akt1 in cell proliferation, cell survival and tumorigenesis and it appears to play a predominant role in hormone receptor-positive breast cancers. Akt3 has primarily been associated with the survival and progression of TNBC. The function of Akt1 and Akt2 in tumor progression and metastasis is debatable. While systemic deletion of Akt isoforms validated Akt1 as a suitable target for cancer therapy in hormone receptor-positive and HER2-enriched breast cancers, it remains to be seen how systemic deletion of Akt1, Akt2 and Akt3 in different subtypes of breast cancer affects breast cancer development and progression. Thus, while Akt remains an important target for breast cancer therapy, a complete understanding of how Akt isoforms contribute to breast cancer is essential.

## Data Availability

Not applicable.

## Abbreviations

**Abbreviation**	**Definition**
AL (A-loop)	Activation loop
CCAR1	Cycle and apoptosis regulator 1
CDK	Cyclin-dependent kinase
CKI	Cyclin-dependent kinase inhibitor
DNA-PK	DNA-dependent protein kinase
EGFR	Epidermal growth factor receptor
EMT	Epithelial-to-mesenchymal transition
ER	Estrogen receptor
ErbB2	Epidermal growth factor receptor 2
ERK	Extracellular signal-regulated kinase
FADD	Fas-associated death domain
HER2	Human epidermal growth factor receptor 2
HM	Hydrophobic motif
MAP1LC3/LC3	Microtubule-associated protein 1 light chain 3
MEF	Mouse embryo fibroblasts
mTOR	Mechanistic target of rapamycin
mTORC1	Mechanistic target of rapamycin complex 1
mTORC2	Mechanistic target of rapamycin complex 2
NF1	Neurofibromin 1
PDK1	Phosphoinositide-dependent kinase 1
PH	Pleckstrin homology
PHLPP	PH domain and leucine-rich repeat protein phosphatase
PI3K	Phosphatidylinositol 3- kinase
PIK3CA	PI3K catalytic subunit alpha
PIP2	Phosphatidylinositol (4,5)-bisphosphate
PIP3	Phosphatidylinositol (3,4,5)-triphosphate
PKB	Protein kinase B
PP2A	Protein phosphatase 2A
PR	Progesterone receptor
PKC	Protein kinase C
PTEN	Phosphatase and tensin homolog deleted on chromosome 10
SETDB1	SET domain bifurcated 1
Skp2	S-phase kinase associated protein 2
TCGA	The cancer genome atlas
TERT	Telomerase reverse transcriptase
TFEB	Transcription factor EB
TIM	TOR-interacting motif
TM	Turn motif
TNBC	Triple-negative breast cancer
TRAF6	Tumor necrosis factor receptor-associated factor 6
UVRAG	Ultraviolet irradiation resistance-associated gene
